# Modulation of Gut Microbiota, Short-Chain Fatty Acid Production, and Inflammatory Cytokine Expression in the Cecum of Porcine Deltacoronavirus-Infected Chicks

**DOI:** 10.3389/fmicb.2020.00897

**Published:** 2020-06-04

**Authors:** Hai-yan Li, Hong-lei Zhang, Fu-jie zhao, Shi-qiong Wang, Zhi-xiang Wang, Zhan-yong Wei

**Affiliations:** ^1^College of Animal Science and Veterinary Medicine, Henan Agricultural University, Zhengzhou, China; ^2^Key Laboratory for Animal-Derived Food Safety of Henan Province, Henan Agricultural University, Zhengzhou, China

**Keywords:** Porcine deltacoronavirus (PDCoV), SPF chick, intestinal microbiota, inflammatory cytokines, short-chain fatty acids

## Abstract

Porcine deltacoronavirus (PDCoV) is a novel swine enteropathogenic coronavirus that causes watery diarrhea and induces proinflammatory cytokine responses in piglets. Our previous research showed that the specific-pathogen-free (SPF) chicks exhibited mild diarrhea and low fecal viral shedding, along with cecum lesions after PDCoV infection. Disturbances in the homeostasis of the gut microbiota have been associated with various diseases. We aimed to explore the effects of PDCoV infection on chick gut microbiota, short-chain fatty acid (SCFAs) production, and inflammatory cytokine expression in chicks, and also to investigate the relationship between gut microbiota and SCFAs or inflammatory cytokine expression of the PDCoV-infected chicks. Results obtained using 16S rRNA sequencing showed that infection with PDCoV strain HNZK-02 significantly altered the composition of chick gut microbiota, with the reduced abundance of *Eisenbergiella* and *Anaerotruncus* genera at 5 days post-inoculation (dpi) (*P* < 0.05), and an increased abundance of *Alistipes* genus at 17 dpi (*P* < 0.05). The production of SCFAs in the cecum of PDCoV HNZK-02–infected chicks, including acetic acid, propionic acid, and butyric acid, decreased in all cases. The expression of inflammatory cytokines (interferon-γ, tumor necrosis factor-α, and interleukin-10) was increased in the cecum tissue and serum of the PDCoV HNZK-02–infected chicks when detected by quantitative real-time polymerase chain reaction and enzyme-linked immunosorbent assay, respectively. Further analysis showed significant correlation between bacterial genera and SCFAs or inflammatory cytokines expression in cecum of the PDCoV infected chicks. These findings might provide new insight into the pathology and physiology of PDCoV in chicks.

## Introduction

Porcine deltacoronavirus (PDCoV) is an enveloped, positive-sense single-stranded RNA virus, with a genome length of approximately 25 kb (Zhang, 2016). This virus was originally found in Hong Kong in 2012 (Woo et al., 2012), and the first outbreak of PDCoV-related diarrhea in swine herds was reported in the United States in 2014 (Wang et al., 2014). As an important enteropathogen in pigs, PDCoV can cause acute diarrhea, vomiting, and dehydration in neonatal piglets (Chen et al., 2014; Hu et al., 2016). Moreover, the infected pigs are characterized by thin and transparent intestinal walls and accumulation of large amounts of yellow fluid in the intestinal lumen (Jung et al., 2015; Li et al., 2019). Further, PDCoV-infected piglets show signs of proinflammatory cytokine responses during acute infection (Jung et al., 2018). PDCoV has caused huge economic losses for the pig industries. Apart from infection of gnotobiotic (Gn) and conventional pigs, PDCoV was also reported to have limited ability to infect Gn calves (Jung et al., 2017). Recent studies showed the susceptibility of specific-pathogen-free (SPF) chicks to PDCoV infection, with the clinical syndrome of mild diarrhea and slight lesions in cecum, which was weaker than that of piglets infected with PDCoV (Liang et al., 2019; Boley et al., 2020). However, the reason why PDCoV infection can cause intestinal damage is still unknown.

The chicken gastrointestinal tract is an important site for immune cell development, which not only regulates gut microbiota, but also maintains extraintestinal immunity. Intestinal commensal microbes play a crucial role in gut homeostasis through mutually beneficial interactions with the host immune system (Deriu et al., 2016). Once the intestinal mucosal barrier and microbiota are destroyed, intestinal inflammation will occur. In turn, the host’s inflammatory response will elicit changes in the microbial community structure (Deriu et al., 2016). The gut microbiota produces various metabolites, including short-chain fatty acids (SCFAs), which can regulate the host antiviral immune response (Budden et al., 2017). Propionate and butyrate have also been reported to induce differentiation of T-regulatory cells, assisting in control of intestinal inflammation (Donohoe et al., 2014; Louis et al., 2014).

In chicks, recent studies have emphasized the key roles of intestinal microbiota in shaping immunity against viral diseases, including avian influenza, Marek’s disease, Newcastle disease (ND), and infectious bursal disease (IBD). These studies not only reflected connections between gut microbiota and distal organs in regulatory functions, but also emphasized the interaction between intestinal microbiota and viral infections and their impacts on immune regulations (Lillehoj and Trout, 1996; Balamurugan and Kataria, 2006; Li, 2007; Chen et al., 2017; Yitbarek et al., 2018). It was reported that different commensal bacteria had their own unique role against viral infection by modulating diverse immune mechanisms (Abt et al., 2012). In the H9N2 avian influenza virus infected chicks, increased levels of interferon gamma (IFN-γ). tumor necrosis factor alpha (TNF-α), and interleukin-17A (IL-17A) led to intestinal microflora dysbiosis (Li H. et al., 2018). However, there are no studies emphasizing how PDCoV affects the gut microbiota in chicks. Thus, in the current study we aimed to observe the intestinal microbiota, SCFAs, and inflammation responses to PDCoV infection in chicks and elucidate the potential associations among them, which will provide the theoretical basis for investigation of the etiology and pathogenesis of PDCoV.

## Materials and Methods

### Virus

The virulent PDCoV strain-HNZK-02 (GenBank accession number MH708123) was isolated and identified by our laboratory and propagated in LLC-porcine kidney (LLC-PK) cells. Passage 5 of this strain was used in current study. Virus propagation and virus titers were performed as described previously (Liang et al., 2019).

### Experimental Infection of SPF Chicks With PDCoV Strain-HNZK-02

The SPF chicks were purchased from Jinan SIPAFAS Poultry Co. Ltd. in Jinan, China. Twenty-four-day-old SPF chicks with similar body weight (about 50 g/chick) were randomly divided into two groups, the control (mock) and PDCoV HNZK-02–infected groups. All chicks were housed in the same facility but in different rooms under biosafety conditions and allowed free access to water and feed during the experiment. Feed was formulated to meet or exceed the National Research Council nutrient requirements for chicks (National Research Council [NRC], 1994; Table 1). Each chick was intragastrically inoculated with PDCoV strain HNZK-02 or Eagle’s Minimum Essential Medium (MEM) with 300 μL/chick (10.5 log10 GE/chick) (*n* = 10/per group).

**TABLE 1:** The composition and nutrients of the basal diet.

**Ingredient**	**Content (%)**	**Chemical composition**	**Content**
Corn	55	CP,%	20.6
Soybean meal	31	ME, Mcal/kg	3
Wheat shorts	3.3	Calcium,%	1
Fish meal	3	Total P,%	0.65
Soybean oil	3.5	Available P,%	0.45
dl-Methionine	0.27	Methionine + cysteine,%	0.9
NaCl	0.27	Lysine,%	1.05
Limestone	1.33	–	–
Calcium phosphate	1.33	–	–
Vitamin-mineral premix^a^	1	–	–

*^*a*^Supplied per kilogram of diet: vitamin A (retinyl acetate), 1,600 IU; cholecalciferol, 300 IU; vitamin E (DL-α-tocopheryl acetate), 10 IU; riboflavin, 3.8 mg; pantothenic acid, 11 mg; niacin, 30 mg; cobalamin, 11 μg; choline chloride, 1,000 mg; biotin, 0.16 mg; folic acid, 0.45 mg; thiamine 1.5 mg; pyridoxine 3.0 mg; Fe, 68 mg; Zn, 50 mg; Mn, 60 mg; I, 0.2 mg; Cu, 8 mg; Se, 0.16 mg.*

### Sample and Tissue Collection

Based on our previous study (Liang et al., 2019), three chicks in each group were selected randomly for necropsy at 5 and 17 dpi, respectively. The cecal contents were collected for analysis of gut microbiota and SCFAs. The cecum tissue was collected for inflammatory cytokine mRNA measurements. The serum was collected for inflammatory cytokine detection by enzyme-linked immunosorbent assay (ELISA).

### Viral RNA Extraction and Quantitative Real-Time Polymerase Chain Reaction

Viral RNA was extracted from cecal content suspensions using the TRizol^TM^ method (Invitrogen, Carlsbad, CA, United States) according to the manufacturer’s instructions. The viral RNA was further reverse transcribed into cDNA. Viral RNA titers were determined using quantitative real-time polymerase chain reaction (qRT-PCR) as reported previously (Liang et al., 2019). The detection limit of the qRT-PCR was 4.6 log10 GE/mL.

### DNA Extraction From the Cecal Content and 16S rRNA Sequencing

Total genomic DNA was extracted from 0.5 g of colonic contents using the FastDNA^®^ SPIN Kit (MP Bio, Santa Ana, CA, United States) following the manufacturer’s guidelines, with the additional glass-bead beating steps on a FastPrep 24 homogenizer (MP Biomedicals, Santa Ana, CA, United States). The final DNA concentration was quantified on a NanoDrop 2000 UV-vis spectrophotometer (Thermo Scientific, Wilmington, DE, United States). The integrity and size of genomic DNA was checked by 1.0% agarose gel electrophoresis. The DNA extracts were sequenced for 16S rRNA using Illumina MiSeq (Illumina, San Diego, CA, United States), targeting the V3–V4 region with barcoded 338F (5′-ACTCCTACGGGAGGCAGCAG-3′) and 806R (5′-GGACTACHVGGGTWTCTAAT-3′) universal primers (Mori et al., 2013), and sequences were processed according to the standard protocols by Majorbio Bio-Pharm Technology Co. Ltd. (Shanghai, China).

### Determination of SCFAs

The SCFAs in cecum were determined using gas chromatography (GC) according to a previously described method (Stewart et al., 2010). The samples were placed on a DB-WAX column (30 m long × 0.53 mm diameter and 1.00 μm film thickness) and were separated by using a TRACE^TM^ 1300 GC with flame ionization detector (FID). The temperature program was 100°C for 0.5 min, then raised to 170°C at 8°C/min and held for 1 min, then raised to 200°C at 40°C/min and held for 2 min. Samples were run with a 30:1 split ratio and a 5.0 mL/min column flow. High-purity hydrogen was used as the carrier gas. The temperatures of the injector and detector were 250 and 270°C, respectively.

### Detection of Inflammatory Cytokine Expression

The levels of expression of the inflammatory cytokines (IFN-γ, TNF-α, and IL-10) in the cecum of SPF chicks were quantified by qRT-PCR. Cecum tissue samples were homogenized by the TISSUELYSER-24 (Jingxin, Shanghai, China). Total RNAs were extracted from the supernatant of the cecum tissue lysate by TRizol^TM^ Reagent Kit (Invitrogen, Carlsbad, CA, United States) and were quantified by NanoDrop 2000 spectrophotometer (Thermo Fisher Scientific, Waltham, MA, United States), then further reverse transcribed into cDNA. The total reaction volume of 20 μL contained 2.0 μL of DNA, 10.0 μL of SYBR Green qPCR Mix (Takara Bio Inc., Tokyo, Japan), 7 μL of H_2_O, and 0.5 μL of each specific primer. Primers were synthesized according to previous reports (Emami et al., 2019) and are shown in Table 2. Reactions were performed under the following conditions: one cycle of preincubated samples at 95°C for 1 min and 39 cycles of amplification samples at 95°C for 5 s, 60°C for 30 s, 72°C for 30 s. Target gene expression of each sample was normalized using glyceraldehyde 3-phosphate dehydrogenase (GAPDH). The relative expression of the target gene for each sample was calculated using the 2^–ΔΔ*Ct*^ method (Livak and Schmittgen, 2001).

**TABLE 2:** The sequences of primers used in this study for real-time RT-PCR.

**Genes**	**Forward primer (5′ to 3′)**	**Reverse primer (5′ to 3′)**	**Amplified size (bp)**
IFN-γ	GCTCCCGATGAACGACTTGA	TGTAAGATGCTGAAGAGTTCATTCG	63
TNF-α	CCCATCCCTGGTCCGTAAC	ATACGAAGTAAAGGCCGTCCC	77
IL-10	CGCTGTCACCGCTTCTTCA	CGTCTCCTTGATCTGCTTGATG	63
GAPDH	CCTAGGATACACAGAGGACCAGGTT	GGTGGAGGAATGGCTGTCA	64

*From Emami et al. (2019).*

The expression levels of the IFN-γ, TNF-α and IL-10 in serum were detected using ELISA Kit from Nanjing Jian cheng Biological Institute (Nanjing, China). Serum samples were obtained and measured as previously described (Huang et al., 2015). Absorbance was read at 450 nm using a microplate spectrophotometer. The inflammatory cytokine concentrations were calculated according to a best-fit standard curve.

### Data Processing and Statistical Analysis

Raw fastq files were demultiplexed, quality-filtered by Trimmomatic, and merged by FLASH (Magoč and Salzberg, 2011). Sequences with ambiguous bases, shorter than 200 bp, and sequences with an average mass less than 25 were removed. The high-quality reads were clustered into operational taxonomic units (OTUs) with 97% similarity cutoff using UPARSE (version 7.11) and used for further analysis of the Venn diagram, alpha diversity indices (Shannon and Simpson), and richness estimators (ACE and Chao). Beta diversity measures were based on weighted-uniFrac distance analysis and shown by principal coordinate analysis (PCoA), which was conducted to assess the relationships among the different groups. LEfSe was applied to recognize which bacterial taxa contribute to the differences between the two groups (Segata et al., 2011), and the minimal threshold of linear discriminant analysis (LDA) was set at 3.5. The difference between specific taxa was analyzed using one-way analysis of variance (ANOVA) followed by *post hoc t-*tests. Data analysis of inflammatory cytokines and SCFAs was performed using GraphPad Prism (version 6.01, La Jolla, CA, United States). A *P* < 0.05 or 0.01 was considered statistically significant or highly significant, which is indicated as follows: ^∗^*P* < 0.05, ^∗∗^*P* < 0.01.

## Results

### Apparent Clinical Characteristics of SPF Chicks Infected With PDCoV

SPF chicks, intragastrically inoculated with PDCoV HNZK-02 at 4 days old, showed mild diarrhea at 5 dpi. PDCoV RNA was detected in the intestinal contents by qRT-PCR at 5 and 17 dpi while non-infected control chicks appeared normal throughout the course of the experiments. PDCoV HNZK-02 infection had no effect on feed consumption compared to controls during the whole experimental period.

### Characteristics of Pyrosequencing Results

**FIGURE 1:**
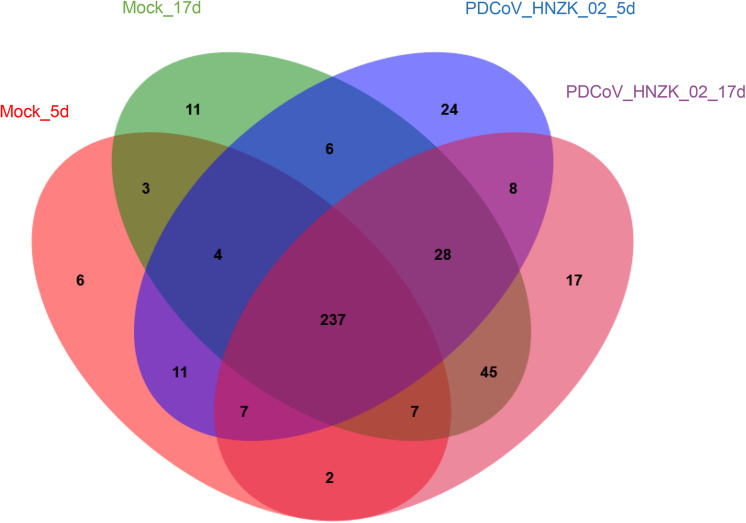
Venn map. Venn map of shared OTUs based on the sequences with more than 97% similarity (*n* = 3). The mock group at 5 dpi (mock-5d), the PDCoV HNZK-02 group at 5 dpi (HNZK-02-5d), the mock at 17 dpi (mock-17d), and the PDCoV HNZK-02 group at 17 dpi (HNZK-02-17d).

A total of 12 cecal concent samples, the control chicks (*n* = 3), and PDCoV HNZK-02 groups (*n* = 3) at 5 and 17 dpi, respectively, were evaluated using 16S rRNA gene sequencing. A total of 663,736 sequences with a median read length of 429 bp (range from 274 to 494 bp) were collected. The total number of unique sequences from the four groups was 416. The sequence and species-level OTUs of each group are shown in Table 3. The Venn diagram showed that 237 OTUs of the total gut microbial richness (416) were shared among all the sequenced samples. Two hundred and fifty-nine OTUs were shared between the samples of the mock and PDCoVHNZK-02 groups with a total of 343 at 5 dpi. Three hundred and seventeen OTUs of the total gut microbial richness (375) were shared between the samples of mock and PDCoV HNZK-02 groups at 17 dpi (Figure 1).

**TABLE 3:** Comparison of phylotype coverage and diversity estimation of the 16S rRNA gene libraries at 97% similarity from the pyrosequencing analysis.

**Group**	**No. of reads**	**No. of OTUs**	**Coverage (%)**	**Richness estimator**		**Diversity index**	
				**ACE (mean ± SE)**	**Chao (mean ± SE)**	**Shannon (mean ± SE)**	**Simpson (mean ± SE)**
Mock_5d	179,564	277	99.95	226.37 ± 2.67^c^	231.41 ± 5.95^b^	3.58 ± 0.16	0.057 ± 0.13
PDCoV HNZK_02_5d	160,137	325	99.93	282.33 ± 0.51^b^	293.29 ± 2.87^a^	3.53 ± 0.11	0.072 ± 0.15
Mock_17d	168,425	341	99.95	303.29 ± 13.75^a,b^	302.96 ± 16.46^a^	3.82 ± 0.06	0.054 ± 0.09
PDCoV HNZK_02_17d	155,610	351	99.93	311.57 ± 7.58^a^	318.37 ± 9.47^a^	3.62 ± 0.06	0.077 ± 0.08

*The operational taxonomic units (OTUs) were defined at a 97% similarity level. The coverage percentage (Good’s) and richness estimators (ACE and Chao) and diversity indices (Shannon and Simpson) were calculated using Good’s method and the mothur program, respectively. In the same column, values with the same superscript letter are not significantly different (*P* > 0.05); those with different superscript letters differ significantly (*P* < 0.05).*

### Alpha and Beta Diversity Analyses

Alpha diversity estimators of community are shown in Table 3. Gut microbial richness, according to the Ace and Chao indexes, was significantly increased in the PDCoV HNZK-02 group when compared with that of the mock group at 5 dpi (*P* < 0.05), while there was no significant difference between the two groups at 17 dpi (*P* > 0.05). In Shannon and Simpson alpha diversity indexes, the differences between the PDCoV HNZK-02 and the mock groups at 5 and 17 dpi did not reach statistical significance (*P* > 0.05).

**FIGURE 2:**
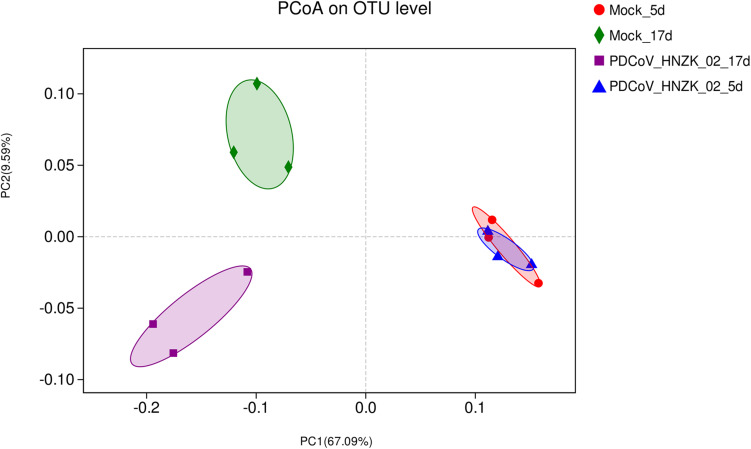
Beta diversity measures in cecal microbiota of chickens (*n* = 3). The scatterplot of the principal coordinate analysis (PCoA) scores shows four different clusters (weighted-uniFrac distance), the blue represents the PDCoV HNZK-02 group at 5 dpi (HNZK-02-5d), the red represents the mock group at 5 dpi (mock-5d), the purple represents the PDCoV HNZK-02 group at 17 dpi (HNZK-02-17d), and the green represents the mock group at 17 dpi (mock-17d).

Beta diversity analysis showed that there were some similarities in the microbial composition between the mock and the PDCoV HNZK-02 groups at 5 dpi. However, the microbial composition from chicks with PDCoV inoculation and the controls could be divided into two different clusters at 17 dpi, indicating that the cecal microbial community structure and composition in the control group were significantly different from those in the PDCoV HNZK-02 group at 17 dpi (Figure 2).

### Difference in Specific Taxa Between PDCoV HNZK-02-Infected Chicks and Controls

**FIGURE 3:**
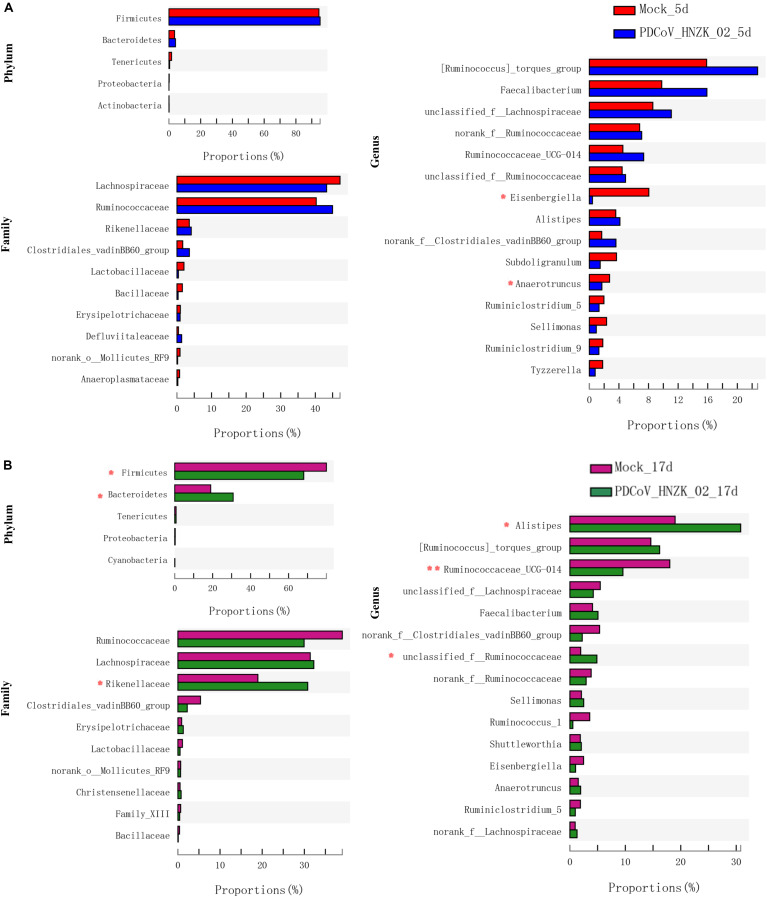
Significant differences analysis in cecal microbiota of chickens. **(A)** Statistical comparison of the relative microbial abundance from phylum to genus between the mock and the PDCoV HNZK-02 groups at 5 dpi (*n* = 3). **(B)** Statistical comparison of the relative microbial abundance from phylum to genus between the mock and the PDCoV HNZK-02 groups at 17 dpi (*n* = 3). **P < 0.01; *P < 0.05.

To further investigate whether PDCoV HNZK-02 infected chicks experienced any significant alteration in gut microbiota, we analyzed the relative abundance of microbiota at the phylum, family, and genus levels. At 5 dpi, genus level analysis showed the taxonomic abundance was altered in the PDCoV HNZK-02 group, which was characterized by lower abundance of *Eisenbergiella* and *Anaerotruncus* (*P* < 0.05). There were no significant differences at the levels of phylum and family between the mock and the PDCoV HNZk-02 groups (Figure 3A). At 17 dpi, the ratio of Firmicutes to Bacteroidetes in the PDCoV HNZk-02 group was lower than that of the mock group. At the family level, the proportion of Rikenellaceac in the chicks inoculated with PDCoV HNZK-02 significantly increased (*P* < 0.05). At the genus level, *Alistipes* and the unclassified f-Ruminococcaceae were significantly increased (*P* < 0.05), whereas the proportion of Ruminococcaceae-UGG-014 was significantly decreased in the PDCoV HNZK-02 group when compared to that of the mock group at 17 dpi (*P* < 0.01) (Figure 3B).

**FIGURE 4:**
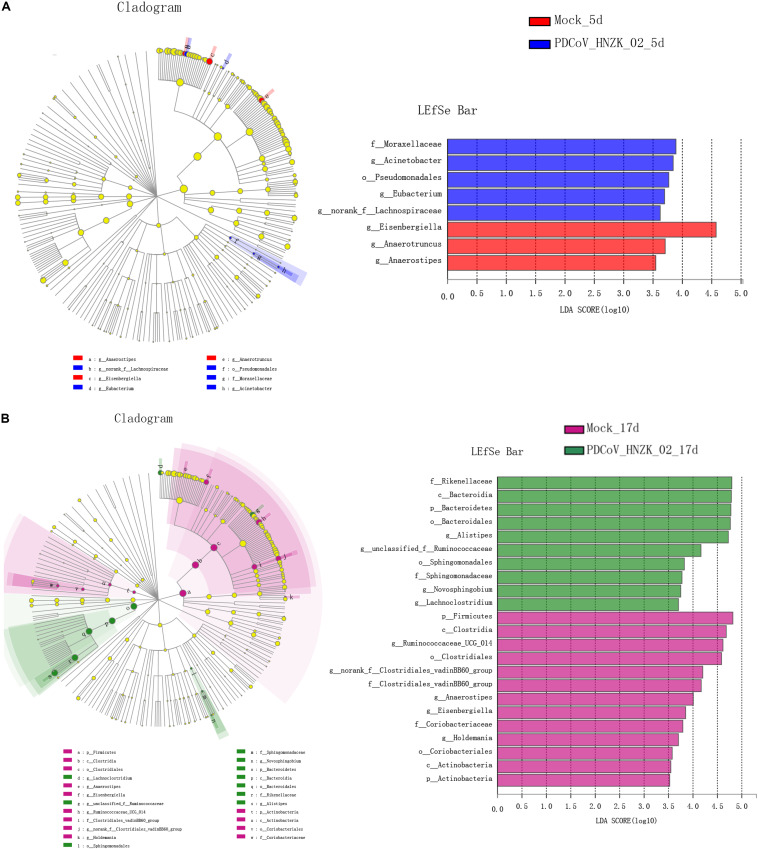
LEfSe analysis in cecal microbiota of chickens. **(A)** LEfSe identifies the taxa with the greatest differences in abundance from phylum to genus between the mock and the PDCoV HNZK-02 groups at 5 dpi (*n* = 3). **(B)** LEfSe identifies the taxa with the greatest differences in abundance from phylum to genus between the mock and the PDCoV HNZK-02 groups at 17 dpi (*n* = 3). Linear discriminant analysis (LDA) effect size (LefSe) analysis showing those abundance from phylum to genus between groups; only taxa of an LDA significant threshold of 3.0 are shown.

The characteristics of bacterial taxonomic abundant in the collected samples were further analyzed using LEfSe algorithm (Figure 4). LEfSe analysis revealed that there existed main 8 and 33 discriminative features from phylum to genus (LDA score >3) between the mock and the PDCoV HNZK-02-inoculated chicks at 5 and 17 dpi (Figures 4A,B), respectively. More specifically, *Eisenbergiella* and *Anaerotruncus* were the most differential microbiota in the mock at 5 dpi. Moraxellaceae, *Acinetobacter*, Pseudomonadates, *Eubacterium*, and *norank-f Lachnospiraceae* in the PDCoV HNZK-02 group increased when compared to that in the mock group. At 17 dpi, we identified 10 microbial biomarkers from the PDCoV HNZK-02 group, including Rikenellaceae, Bacteroidia, Bacteroidetes, Bacteroidales, *Alistipes*, unclassified-f-Ruminocococcaceae, Sphingomonadaceae, *Novosphingobium*, and *Lachnoclostridium*, which could be distinguished from the mock group. 13 clades were more abundant in the mock group than that in the PDCoV HNZK-02 group, including Firmicutes, Clostridia, *Ruminocococaceae-UCG-014*, Clostridia, *norank-f-Clostridiales-vadinBB60-group*, Clostridiales-vadinBB60-group, *Anaerostipe*, *Eisenbergiella*, Coriobacteriaceae, *Holdemania*, Coriobacteriales, Actinobacteria, and Actinobacteria. There were bigger differences on microbiota compositions between the early and later stages of PDCoV infection (at 5 and 17 dpi, respectively).

### SCFA Concentration in Cecal Content of Chicks Inoculated With PDCoV

**FIGURE 5:**
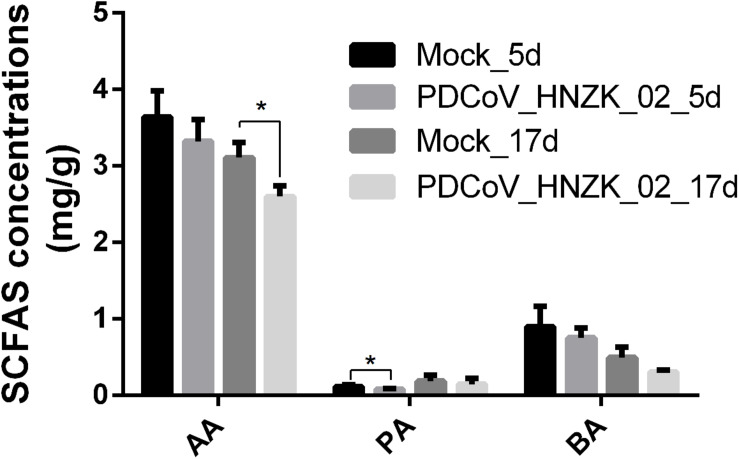
Effects of PDCoV HNZK-02 infection on SCFAs in the cecum of chickens. The SCFAs level of the cecal content among the mock and the PDCoV HNZK-02 groups at 5 and 17 dpi (*n* = 3). AA, acetic acid; dpi, days post inoculation; BA, butyric acid; PA, propionic acid.

Compared to the control, there was a decreasing trend in the amounts of acetic acid (AA), propionic acid (PA), and butyric acid (BA) in the PDCoV HNZK-02 group at 5 and 17 dpi. Furthermore, PA and AA levels in the PDCoV HNZK-02 group were greatly decreased at 5 and 17 dpi, respectively (*P* < 0.05) (Figure 5).

### PDCoV HNZK-02 Infection Promotes the Expression of Inflammatory Cytokines in Chicks

**FIGURE 6:**
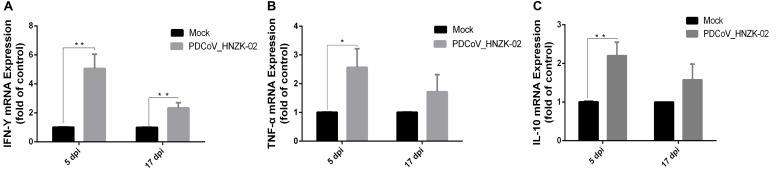
PDCoV HNZK-02 infection promotes inflammatory factors (IFN-γ, TNF-α and IL-10) expression in mRNA levels in the cecum of SPF chickens (*n* = 3). **(A)** IFN-γ expression in cecum at 5 and 17 dpi. **(B)** TNF-α expression in cecum at 5 and 17 dpi. **(C)** IL-10 expression in cecum at 5 and 17 dpi. **P < 0.01; *P < 0.05. dpi, days post inoculation; IFN-γ, interferon gamma; IL-10, interleukin 10;TNF-α, tumor necrosis factor alpha.

As shown in Figure 6, the expression of IFN-γ (*P* < 0.01), TNF-α (*P* < 0.05) and IL-10 (*P* < 0.01) was significantly upregulated at 5 dpi compared with that in the mock chicks (Figures 6A–C). Furthermore, IFN-γ expression was significantly upregulated (Figure 6A) at 17 dpi (*P* < 0.01).

**FIGURE 7:**
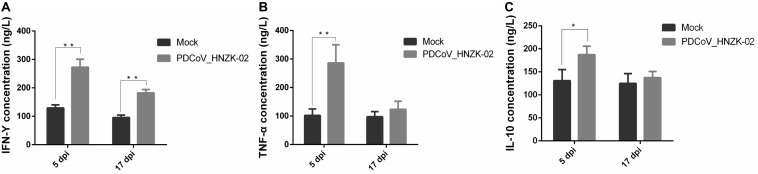
PDCoV HNZK-02 infection increases the expression of inflammatory factors (IFN-γ, TNF-α, and IL-10) in the serum of chickens. **(A)** IFN-γ expression in serum at 5 and 17 dpi (*n* = 3). **(B)** TNF-α expression in serum at 5 and 17 dpi. **(C)** IL-10 expression in serum at 5 and 17 dpi. **P < 0.01, *P < 0.05. dpi, days post inoculation; IFN-γ, interferon gamma; IL-10, interleukin 10; TNF-α, tumor necrosis factor alpha.

To evaluate if PDCoV infection could affect IFN-γ, TFN-α, and IL-10 secretion in serum, the concentrations of IFN-γ, TFN-α, and IL-10 in serum were tested with ELISA kits. Our results showed that the changes of the inflammatory cytokines in serum were similar with those in the cecum tissue. The levels of IFN-γ and TNF-α were approximately two and three times as high as in the PDCoV HNZK-02 group when compared to that of the mock group at 5 dpi (*P* < 0.01). The level of IL-10 in the PDCoV infection group was also increased at 5 dpi (*P* < 0.05). The serum levels of IFN-γ in the PDCoV-infected chicks were higher than in the mock group at 17 dpi (*P* < 0.01), and no significant differences were observed for the secretion of TNF-α and IL-10 between the mock and the PDCoV HNZK-02 groups at 17 dpi (Figures 7A–C).

### Relationship Between Microbial Signatures and the Levels of SCFAs or the Expression of Inflammatory Cytokines in Chicks Infected With PDCoV

**FIGURE 8:**
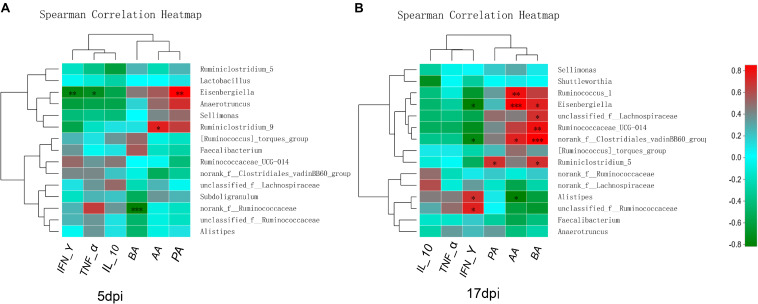
Relationships between bacterial composition and SCFAs or inflammatory factors in cecum. Heatmap of the correlation analysis was conducted between the top 15 bacterial genera and the environmental factors. **(A)** Correlation heatmap at 5 dpi. **(B)** Correlation heatmap at 17 dpi. Red indicates a positive correlation, while green indicates a negative correlation. The deeper color means the greater correlation. *0.01 < *P* ≤ 0.05, **0.001 < *P* ≤ 0.01, ***P ≤ 0.001. AA, acetic acid; BA, butyric acid; IFN-γ, interferon gamma; IL-10, interleukin 10; PA, propionic acid; TNF-α, tumor necrosis factor alpha.

Spearman’s correlation analysis was conducted between the top 15 bacterial genera and the environmental factors (SCFAs, proinflammatory cytokines), and was directly reflected by a heatmap (Figure 8). The threshold | *R*| > 0.8 was considered as having a correlation. The results indicated that *Eisenbergiella* was negatively correlated with TNF-α (*P* < 0.05) and IFN-γ (*P* < 0.01) expression levels, and was positively correlated with PA (*P* < 0.01) at 5 dpi (Figure 8A). The *Ruminiclostridium_9* also showed a positively correlation with the IL-10 expression (*P* < 0.05), while Norank_f_ Ruminococcaceae was negatively correlated with BA (*P* < 0.001). At 17 dpi (Figure 8B), *Alistipes* and unclassified_f_ Ruminococcaceae showed an extremely significant positive correlation with IFN-γ secretion (*P* < 0.05), but Eisenbergiella and Norank_f_Clostridiales_vadinbb60_group were negatively correlated with it (*P* < 0.05). The level of PA was associated with the increase in the abundance of Ruminiclostridium_5 (*P* < 0.05). *Ruminococcus* and *Eisenbergiella* were positively correlated with AA (*P* < 0.05), while *Alistipes* showed a negative relationship with it (*P* < 0.05). Eisenbergiella, unclassified_f_Lachnospiraceae, Ruminococcaceae_UCG-014, Norank_f_Clostridiales_vadinbb60_group, and Ruminiclostr idium_5 were positively correlated with BA.

## Discussion

Gut microflora play an important role in shaping immunity against viral diseases in host. Disruption of microbial homeostasis is associated with a variety of pathological states, which helps the establishment of acute viral infections in chickens (Abaidullah et al., 2019). In this study, the infection model of PDCoV was developed on SPF chicks. Our results demonstrated similar clinical diseases, and the dynamics of the virus shedding in cecum were observed in all SPF chicks inoculated with the PDCoV HNZK-02 strains when compared with our previous experiment (Liang et al., 2019). The influence of PDCoV infection on cecum microbiota of SPF chicken was evaluated, PDCoV HNZK-02 infection significantly altered the gut microbiota composition and decreased SCFAs products in chicks’ cecum. Simultaneously, the expression levels of inflammatory cytokines (IFN-γ, IL-10, and TNF-α) in serum and cecum tissue were significantly increased, resulting in the inflammatory response in the chicken. The analysis showed a significant correlation between bacterial genera and SCFAs or inflammatory cytokines.

The diversity of indigenous intestinal microbiota is one of the key factors in resisting the colonization of invading pathogens (Keesing et al., 2010). Several studies have reported that the diarrhea-relating coronaviruses could influence the intestinal microbiota diversity in pigs (Liu et al., 2015; Song et al., 2017; Huang et al., 2018; Tan et al., 2019). Indeed, many viral agents have been shown to alter the intestinal microbiota in chickens, such as AIV (Yitbarek et al., 2018), Marek’s disease virus (MDV) (Lillehoj and Trout, 1996), NDV (Li, 2007), and IBDV (Li L. et al., 2018). In PDCoV-infected chicks, the species richness indices were increased at 5 dpi, and there were no significant changes in the overall α-diversity of cecal microbiota at 17 dpi. It is possible that the reduced immune response may account for the change of microbial diversity. From the PCoA results, microbiota structure in the PDCoV infected group was clearly distinguished from that of the mock group at 17 dpi, which also reflected SPF chicks’ resistance to PDCoV.

From microbial community profiling, the difference between the groups was analyzed at the phylum, family, and genus level. *Eisenbergiella* and *Anaerotruncus* genera were significantly decreased in the PDCoV HNZK-02 at 5 dpi, indicating that PDCoV HNZK-02 infection had a marked influence on chicks’ cecal microbiota. According to previous studies, the decrease of the ratio of Firmicutes-to-Bacteroidetes was observed in mice with diabetes (Wen et al., 2008) and in some patients with Crohn’s disease and ulcerative colitis (Frank et al., 2007). In our results, a decreased ratio of Firmicutes-to-Bacteroidetes occurred in the PDCoV HNZK-02 group at 17 dpi. More remarkably, the *Alistipes*, considered an opportunistic pathogen (Pandit et al., 2018; Zhang et al., 2018), was significantly increased in the PDCoV HNZK-02 group at 17 dpi. We used linear discriminant analysis (LDA) of effect size (LEfSe) to confirm the taxa that most likely explains the differences between the PDCoV HNZK-02 and mock groups. These findings provide compelling evidence that PDCoV HNZK-02 induced microbiota imbalance and showed that recovery was difficult in a short time period after PDCoV HNZK-02 infection.

Production of SCFAs, as the end products of protein and carbohydrate fermentation (Cummings and Macfarlane, 1991), can easily be affected by the status of the gut microbiota (Lloyd-Price et al., 2019). Studies have shown that *Lactobacillus salivarius* and *L. agilis* could increase propionate and butyrate contents in cecum of chicks (Meimandipour et al., 2010). The changes in butyrate and propionate can influence intestinal physiology and immune function, while acetate can alter lipid metabolism (Macfarlane and Macfarlane, 2011). Our data showed that the changes of gut microbiota composition contributed to the decreased SCFAs, which reduced this protective effect of SCFA in the cecum. However, the interaction of gut microbiota with SCFAs to respond to PDCoV infection needs further studies.

Inflammatory immune responses in the gut can alter the gut luminal environment in a way that favors dysbiosis (Winter et al., 2013). Chicks with dysbiosis are more prone to acute viral infection (Abaidullah et al., 2019), which may in turn further affect the pathogen infectivity. *Eisenbergiella* is strongly correlated with increased levels of TNF-α and IFN-γ, which help in modulating the functional activities of the cells of the immune system (Liu et al., 2010), and to block virus replication and prevent clinical disease (Bradley, 2008; Praveena et al., 2010). This may be the reason that *Eisenbergiella* was decreased in the PDCoV HNZK-02 group at 5 dpi. Moreover, the observation is similar to the increased proinflammatory (TNF-α) cytokine responses of 10-day-old Gn pigs to acute PDCoV infection (Jung et al., 2018). *Alistipes* was strong positively correlated with IFN-γ levels, and the numbers of Ruminococcaceae_UCG-014 were strong negatively correlated with IFN-γ levels, indicating that these bacteria can be tolerated, with IFN-γ responses benefiting for those genera of bacteria. The anti−inflammation activity could be explained in the study regarding to the administration of Norank_f_Lachnospiraceae in gut, and was related to the IL−10 cytokine (Olszak et al., 2014). The results showed the change of microbiota in the cecum was related to intestinal inflammation (directly or indirectly, positively or negatively).

## Conclusion

In conclusion, the present study revealed that obvious changes were found in the microbial community of chicks infected with PDCoV HNZK-02. PDCoV-inoculated chicks showed a decrease in *Eisenbergiella* and *Anaerotruncus* populations at 5 dpi and an increase in pathogenetic *Alistipes* at 17 dpi with a reduction in SCFAs production. The changes in the microbial community may be related to changes in inflammatory cytokine expression. These results provide new insights into the pathophysiology of chicks infected with PDCoV.

## Data Availability Statement

The obtained raw sequencing reads have been added to the NCBI SRA page (Accession Number: PRJNA558384). Other data generated during the study are included in this article.

## Ethics Statement

The experimental procedures were approved by the Laboratory Animals Ethics Committee at Hennan Qixiang Biological Technology Co. Ltd. (HNQX-2018-06), and all husbandry practices and euthanasia were performed with full consideration of animal welfare.

## Author Contributions

HL and HZ performed the experiments. HL, FZ, and SW performed statistical analyses and wrote the manuscript. Z-xW and Z-yW designed the study and revised the manuscript. All authors read and approved the final manuscript.

## Conflict of Interest

The authors declare that the research was conducted in the absence of any commercial or financial relationships that could be construed as a potential conflict of interest.

## Abbreviations

AA: acetic acid
BA: butyric acid
dpi: days post inoculation
ELISA: enzyme-linked immunosorbent assay
GC: gas chromatography
Gn: gnotobiotic
IBD: infectious bursal disease
IFN- γ: interferon gamma
IL-10: interleukin 10
LDA: linear discriminant analysis
LEFSe: linear discriminant analysis effect size
LLC-PK: LLC-porcine kidney
OTUs: operational taxonomic units
PA: propionic acid
PDCoV: porcine deltacoronavirus
PCoA: principal coordinate analysis
SPF: the specific pathogen free
SCFAs: short-chain fatty acid
TNF- α: tumor necrosis factor alpha
